# Increased Expression of Matrix Metalloproteinase-1 and 3 in Remission Patients of Steroid-Dependent Ulcerative Colitis

**DOI:** 10.4021/gr2010.05.208w

**Published:** 2010-05-20

**Authors:** Zhi Yong Wang, Bing Feng Qiu

**Affiliations:** a Department of Digestive Diseases, Affiliated Hospital of Hangzhou Normal University (the Second People’s Hospital of Hangzhou City), Hangzhou 310015, Zhejiang Province, China

**Keywords:** Ulcerative colitis, Metalloproteinase, Steroid-dependent

## Abstract

**Background:**

This study was to investigate the pathological significance of protein expression of matrix metalloproteinase-1 (MMP1) and matrix metalloproteinase-3 (MMP3) in colon tissues of remission patients of steroid-dependent uncreative colitis (SDUC).

**Methods:**

To test the possible involvement of MMP-1 and MMP-3 in SDUC, Western- blot and immunohistochemistry were applied to examine the protein expression of MMP-1 and MMP-3 in the colonic healing region from 10 remission patients with SDUC as well as from 10 remission patients with non-SDUC. Ten specimens from normal colon tissues were used as controls.

**Results:**

Compared with the control group, the protein expression of MMP-1 and MMP-3 from non-SDUC remission patients slightly increased (p > 0.05), in contrast, those from SDUC patients significantly increased (p < 0.01).

**Conclusions:**

Over-expression of MMP-1 and MMP-3 play a critical role in the pathogenesis of SDUC.

## Introduction

The etiology of ulcerative colitis (UC) is unclear, the lesions of UC mostly locate in the mucosa and submucosa of colorectal region with chronic unspecific inflammation. Though the steroid is the key agent commonly used for remission of UC, the long-term administration of corticosteroid may reduce its efficacy and incur much side-effects, therefore, currently the steroid is not recommended for the maintenance therapy of UC [1]. Approximately, 20% of UC patients are steroid-dependent ulcerative colitis (SDUC), SDUC is characterized by effective response to corticosteroid (CS) initially but followed by relapse of symptoms if dosage reduction or withdrawal of CS. Usually, several courses of treatment are needed to control the symptoms and achieve remission [2]. Undoubtedly, to elucidate the pathogenesis of SDUC is of utmost importance for overcome this illness. At present, it is generally accepted that the imbalance of synthesis and degradation of extracellular matrix (ECM) is an essential factor in the ulcer formation of UC, during this process, the matrix metalloproteinases (MMPs) play important roles.

MMPs belong to a family of zinc-dependent endopeptidase, which are mainly produced and secreted by connective tissue, endothelial cells, mononuclear macrophages, neutrophils and tumor cells. The MMPs participate in the degradation of ECM components [3]. Many animal and clinical studies showed that in the UC lesion area, several MMPs express increasingly, however, their expression are relatively low in the normal area [4-7], all these evidences indicate that MMPs play key roles in the onset of UC. Within the MMP family, MMP-1 and MMP-3 participate the tissue repair, and play roles in the UC occurrence [8]. However, to our knowledge, there is no report regarding whether the MMP-1 and MMP-3 are associated with the onset of SDUC. In this study, we determined the expression of MMP-1 and MMP-3 in SDUC, in order to investigate their roles in SDUC.

## Patients and Methods

### Patients

Twenty UC patients were from inpatient or outpatient department in our hospital from July 2006 to December 2008. All cases meet the UC diagnostic criteria amended in National Ulcerative Colitis Conference (Chengdu, China) in the year of 2000. Among these 20 cases, 10 non-SDUC patients were controlled effectively by sulfasalazine (SASP) treatment; 10 SDUC patients were treated with prednisone as maintenance therapy. All 20 UC patients achieved remission after treatments. Ten cases from healthy group were used as controls.

This study was approved by the Hospital Ethical Committee, and informed consent was obtained from each patient.

### Reagents

Monoclonal anti-MMP-1 and MMP-3 antibodies were purchased from Santa Cruz (Santa Cruz Biotechnology, Santa Cruz, California, USA). Monoclonal anti-glyceraldehyde phosphate dehydrogenase (GAPDH) antibody was from Kangcheng company (Shanghai, China). Horseradish peroxides (HRP) goat anti-mouse antibody tag was from Jingmei Biotechnology Company (Shanghai, China). Marker for Western blot was purchased from New England Biolabs Inc (Shanghai, China). Electrogenerated chemiluminescence (ECL) kit was purchased from Pierce Company (USA). Immunohistochemistry kit was purchased from Boshide Company (Wuhan, China).

### Sample collection and processing

Specimens from 20 cases of UC patients were taken from multiple points (8-10 points per case) in healing region of colon based on the previous colonoscopy reports. The control samples were taken from 8-10 points in sigmoid colon in each case. Partial samples were frozen immediately in liquid nitrogen and stored at -80 °C, the rest were fixed in 10% neutral formalin.

### Western blot

Western blot was used to detect the expression of MMP-1 and MMP-3. Firstly, total protein was extracted from specimens. The protein sample was boiled for 5 min at 95 - 100 °C and resolved in 10% SDS polyacrylamide gel (running for 2.5 h). The gel was then transferred to a membrane, followed by incubation of the membrane with primary antibody solution, 4°C overnight. After washing, the membrane was incubated with secondary antibody for 1h at room temperature. For the last step, the membrane was washed and detected by ECL. The same procedure was repeated using the same membrane except using anti-GAPDH as primary antibody (1:5000 dilution) for internal standard. The bands were analyzed using Biology Electrophoresis Analysis System FR-980, which can automatically read and record the value of each band. The ratio of sample to internal standard was used for statistic analysis.

### Immunohistochemical staining (Polymeric Method)

The sample sections were deparaffinized and hydrated, followed by incubation with 0.3% H_2_O_2_ in PBS for 10 minutes at 37 °C, then rinsed in 0.01 M PBS (PH 7.35 - 7.4) for 3 times (5 min each). After applied to heat antigen retrieval, the sections were rinsed in 0.01M PBS (PH 7.35 - 7.4) for 3 times (5 min each), then were incubated in 5% goat serum for 20 min at 37 °C to block non-specific bindings. The primary antibody was applied to the sections for 1 hour at 37 °C, then rinsed 3 times (5 min each) in 0.01M PBS (PH 7.35-7.4), incubated with HR coupled secondary antibody for 30 min at 37 °C, and rinsed in 0.01M PBS (PH 7.35 - 7.4) for 3 times (5 min each), followed by incubation with DAB solution for 10 min. Finally, sections were counterstained with hematoxylin for 5 - 10 min. For the control, PBS (pH 7.4) was applied to replace primary antibody.

### Semiquantitative scoring of MMP-1 and MMP-3 expression

For MMP-1 and MMP-3 expression evaluation, a semiquantitative scoring system was used according to the grading immunostaining patterns. Five high-power fields (400x) were randomly chosen for each slide. The percentage of stained cells was scored on a semiquantitative scale of 0-4, using the following criteria: 0, 0%; 1, less than 25% for the stained cells; 2, 25-50%; 3, 50-75%; 4, more than 75%. The intensity of immunostaining was scored as: 0, no staining; 1, weak; 2, moderate; 3, intense. The final immunohistochemical score results were derived from multiplying the percentage score by the staining intensity score, with minimum score of 0 and maximum score of 12.

### Statistical analysis

The software SPSS16.0 (SPSS, Chicago, USA) was used for statistical analysis. The values were expressed as mean ± standard deviation (x ± SD). The statistical differences among multiple group data were determined by one-way ANOVA. A non parametric analysis was used to compare the immunostaining scores. A p < 0.5 was considered statistically significant.

## Results

### MMP-1 and MMP-3 protein expression in colonic tissue

**Figure 1:**
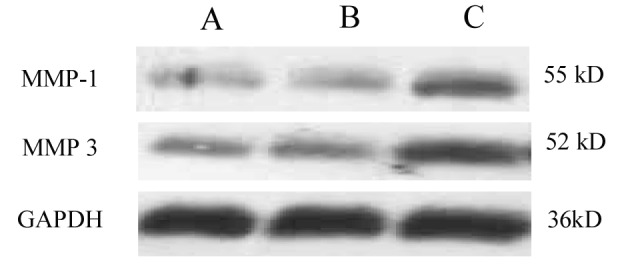
Western blot of MMP-1 and MMP-3 protein expression. Compared with control, MMP-1 and MMP-3 protein expression showed slight increase in non-SDUC patients, but significantly increased in the SDUC group. A, Control; B, non-SDUC group; C, SDUC group.

**Figure 2:**
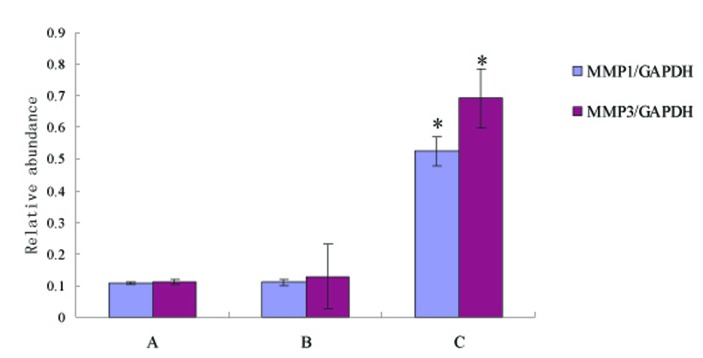
Compared with control, MMP-1 and MMP-3 protein expression showed slight increase in non-SDUC patients (p > 0.05), but significantly increased in the SDUC group (p < 0.01). A, Control; B, non-SDUC group; C, SDUC group. * P < 0.01.

Compared with control, MMP-1 and MMP-3 protein expression were not increased in the healing region of colon in the group of non-SDUC patients (p > 0.05), however, significant increase of the proteins was detected in the SDUC group (p < 0.01) (Fig. 1, 2).

### Immunohistochemical staining of MMP-1 and MMP-3 protein in colon

**Figure 3:**
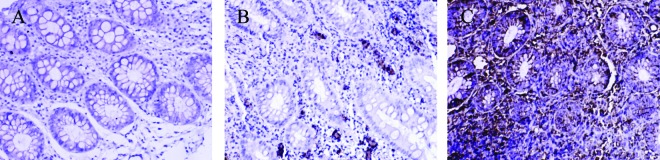
Immunohistochemical staining of MMP-1 protein in colonic tissue. There were no MMP-1 positive staining cells were found in control group; a small amount of MMP-1 positive staining cells were presented in non-SDUC group and were mainly located in glandular regions of colon; a large number of MMP-1 positive staining cells were shown in the SDUC group. A, Control; B, non-SDUC group; C, SDUC group. Original ×400.

**Figure 4:**
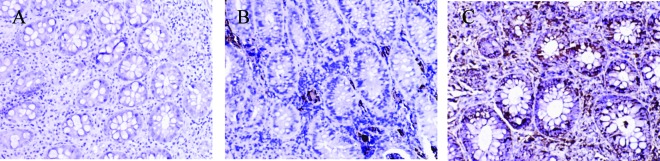
Immunohistochemical staining of MMP-3 protein in colonic tissue. There were no MMP-3 positive staining cells were found in control group; a small amount of MMP-3 positive staining cells were presented in non-SDUC group and were mainly located in glandular regions of colon; a large number of MMP-3 positive staining cells were shown in the SDUC group. A, Control; B, non-SDUC group; C, SDUC group. Original ×400.

No MMP-1 and MMP-3 positive staining cells were found in control group; a small amount of MMP-1 and MMP-3 positive staining cells were presented in non-SDUC group and were mainly located in glandular regions of colon; a large number of MMP-1 (Fig. 3) and MMP-3 positive staining cells were shown in the SDUC group (Fig. 4).

### Semiquantitative assay of MMP-1 and MMP-3 expression

The semiquantitative analysis showed that the expressions of MMP-1 and MMP-3 in SDUC group were significantly higher that of non-SDUC group (P < 0.5).

## Discussion

The steroid dependency of ulcerative colitis is one of the causes of refractoriness in ulcerative colitis. To date, the mechanisms of this disease are not well understood.

Recently, the role of MMPs in pathogenesis of UC has brought more attentions. MMPs participate both the enteric impairment and repair, this was confirmed in an experiment using human fetal intestine culture model, in which the poke week mitogen (PWM) can induce the T lymphocytes to produce MMPs, and the latter impair the intestinal mucosal tissue, presented with epithelial removal and villi disappearance [9].

According to the structure and substrate specificities, the MMPs can be divided into six groups, namely, collagenase, gelatinase, interstitial lysin, elastase, membrane protease and other types of MMP [10]. The evidences have indicated that over-expression and increased activities of MMPs could lead to injury and inflammation in colonic mucosa [11]. Among these MMPs, the MMP-1 and MMP-3, located in fibroblast and macrophages, are playing outstanding roles; they impair the intestinal mucosa after being activated by certain inflammatory factors, such as tumor necrosis factor-α, nuclear factor- κβ, pro-inflammatory interleukins [8].

Within the MMP family, MMP-1 is also known as an interstitial collagenase and mainly acts in degradation of collagen I, II, III, IV and proteoglycans, which renders them sensitive to gelatinase; while the MMP-3 belongs to the family of mesenchymal lysine, it can degrade most of the ECM components and activate other MMPs [12]. Previous studies have reported that the MMP activities significantly increased in both UC and Crohn’s disease [13]. Further studies have also reveled that mRNA expression of MMP-1 and MMP-3 correlates the degree of inflammation in UC patients, and the level of MMP-1 and MMP-3 mRNA expression in inflicted region is 15-fold higher than that in normal colonic mucosa, indicating that over-expression of MMP-1 and MMP-3 in UC patients plays important roles in tissue injury and repairing processes [4, 8].

In this study, we have demonstrated that the protein expression of MMP-1 and MMP-3 significantly increased in healing region of colon tissue in SDUC remission patients but not in non-SDUC remission patients, suggesting that over-expression of MMP-1 and MMP-3 is not only a key factor in the pathogenesis of UC, but also a critical feature in steroid-dependency of UC. Further investigations on this issue are of great significance in clarifying the mechanisms of steroid-dependency in SDUC.
